# Dr. Powell to Go to the International Congress

**Published:** 1887-02

**Authors:** 


### DR. POWELL TO GO TO THE INTERNATIONAL CONGRESS.

We are pleased to announce that our Senior Editor, Dr. T. S. Powell, President of the Southern Medical College and Professor of Obstetrics and Gynæcology in that Institution, has been appointed Vice-President of the Gynæcological Section of the International Medical Congress. If there ever was an occasion illustrative of the fact that a man honors himself by honoring another, this is unmistakably one. The appointment is a good one, and we congratulate Prof. De Laskie Miller, of Chicago, the able President of the Section, in securing so able a man as the vice-president of his Section. E. v. G.

